# Assessment of the development of assisted reproductive technology in Liaoning province of China, from 2012 to 2016

**DOI:** 10.1186/s12913-018-3585-9

**Published:** 2018-11-20

**Authors:** Yuan-Yuan Fang, Qi-Jun Wu, Tie-Ning Zhang, Tian-Ren Wang, Zi-Qi Shen, Jiao Jiao, Xiao-Guang Shao, Peng Xu, Shuai-Shuai Guo, Yi-Ming Zhou, Xiu-Xia Wang, Da Li

**Affiliations:** 1 Center of Reproductive Medicine, Department of Obstetrics and Gynecology, Shengjing Hospital of China Medical University, Shenyang, 110004 China; 2 Department of Clinical Epidemiology, Shengjing Hospital of China Medical University, Shenyang, 110004 China; 3 Department of Pediatrics, Shengjing Hospital of China Medical University, Shenyang, 110004 China; 4 Department of Obstetrics, Gynecology, and Reproductive Sciences, Yale School of Medicine, New Haven, CT 06520 USA; 5 Reproductive and Genetic Medicine Center, Dalian Municipal Women’s and Children’s Medical Center, Dalian, 116031 China; 6 Center of Human Reproduction and Genetics, Jinghua Hospital, Shenyang, 110000 China; 7 Center of Reproductive Medicine, Shenyang Women’s and Children’s Hospital, Shenyang, 110011 China; 8 Department of Medicine, Brigham and Women’s Hospital, Harvard Institutes of Medicine, Harvard Medical School, Boston, MA 02115 USA

**Keywords:** Assisted reproduction techniques, In vitro fertilization, Time trend, Liaoning province, China

## Abstract

**Background:**

The development of assisted reproduction techniques (ART) has resulted in rapid advances in the treatment of infertility. However, a systematic assessment of ART and its processes and outcomes in China has never been carried out. The goal of this study was to assess the features of ART status from 2012 to 2016 in clinics and in vitro fertilization (IVF) laboratories in Liaoning, the largest IVF province in the northeast of China.

**Methods:**

Data from Jan 1, 2012 to Dec 31, 2016 was retrieved from the assisted reproductive certificate registry of Liaoning province. We extracted data from: i) fresh and thawed cycles; ii) donor sperm and donor egg cycles; iii) intrauterine insemination with husband semen and donor semen (AIH and AID).

**Results:**

We showed that: (i) there has been a significant increase in the number of IVF fresh and thawed cycles, and the proportion of cases of primary infertility and secondary infertility has decreased and increased, respectively; (ii) standard long GnRH agonist was the major ovarian stimulation protocol. During the observation period, increasing trends in the use of GnRH antagonists, mild stimulation, and natural cycles were observed; (iii) significant differences in the number of cycles, number of retrieved oocytes, fertilization rates, implantation rates, and sex ratio were noticed between conventional IVF and intracytoplasmic sperm injection; (iv) significant differences in age at treatment for infertility, number of cycles, and ectopic pregnancy rates were noticed between donor sperm cycles and donor egg cycles; (v) significant differences in number of thawed cycles, number of thawed embryos, embryo recovery rates, implantation rates, and clinical pregnancy rates were noticed between day 3 and day 5 embryos; (vi) significant differences in age at treatment for infertility, number of cycles, clinical pregnancy rates, ectopic pregnancy rates, and live birth ratio were noticed between AIH and AID.

**Conclusions:**

ART in Liaoning province has undergone substantial development from 2012 to 2016 in clinics and IVF laboratories. This presentation of detailed ART data will provide researchers, policy makers, and potential ART users a rich source of information about IVF characteristics in the northeast of China.

## Background

Infertility is estimated to affect between 8 and 12% of women of child-bearing age worldwide, and has become a major health burden over the last few decades [1]. Strikingly, the development of assisted reproduction techniques (ART) has resulted in rapid advances in the treatment of infertility. As a result, ART in mainland China has experienced a tremendous evolution since the first in vitro fertilization (IVF) infant was born in 1988 [2], partly reflected by the rapidly increasing number of ART centers and IVF cycles, along with increasing trend of clinical pregnancy rates.

Despite the rapidly growing popularity of ART and IVF, a systematic assessment of ART and its processes and outcomes in China has never been carried out. It is well-known that ART success rates fluctuate due to different patient and treatment factors, such as age, infertility causes, ovarian reserve, ovarian stimulation protocol, type of reproductive technology used such as conventional IVF or intracytoplasmic sperm injection (ICSI), and history of previous pregnancy or ART cycles [3]. Without an accurate data analysis, it is difficult to understand the current status of reproductive medicine in China. In Liaoning, the largest province in the northeast of China, more than 20,000 ART cycles occurred in 2016. Herein, we address these aforementioned questions by evaluating the ART data from Liaoning province 2012 to 2016. The data presented in this report will provide researchers, policy makers, and potential ART users a rich source of information about IVF characteristics in the northeast of China.

## Methods

### Study population and data source

The Center of Reproductive Medicine in Shengjing Hospital of China Medical University is the quality control center of human assisted reproductive technology in Liaoning province. The control center covers all 14 cities of the province (Shenyang, Dalian, Anshan, Fushun, Benxi, Dandong, Jinzhou, Yingkou, Fuxin, Liaoyang, Panjin, Tieling, Chaoyang, and Huludao), with approximately 42 million inhabitants [4, 5]. Data from Jan 1, 2012 to Dec 31, 2016 were retrieved from the assisted reproductive certificate registry of Liaoning province, which is maintained by Shengjing Hospital of China Medical University.

### Data collection and analysis

Provincial and city surveillance networks as well as clinical expert groups were established to undertake the data collection. For the purpose of analysis, the following parameters from each Center of Reproductive Medicine were identified: a) number of fresh, thawed, AIH, and AID cycles; b) age at treatment for infertility, classification of infertility (primary or secondary), and causes (female, male, combined, genetic, and unexplained factors); c) the ovarian stimulation protocol was divided into super long GnRH agonist, standard long GnRH agonist, short GnRH agonist, GnRH antagonist, mild stimulation, and natural cycle. To qualify the patients’ response to ovarian stimulation, we used the ESHRE consensus on the definition of ‘poor response’ to ovarian stimulation for in vitro fertilization, the Bologna criteria (2011) [6]. Patients diagnosed as poor ovarian respone (POR) can accept mild stimulation and natural cycles. The percentage of different protocols, number of retrieved oocytes, implantation rates, clinical pregnancy rates, early miscarriage rates, ectopic pregnancy rates, multiple pregnancy rates, and average dose of gonadotropin for each item were calculated; d) conventional IVF and ICSI. The number of cycles, number of retrieved oocytes, fertilization rates, cleavage rates, implantation rates, sex ratio, live birth rates, and fetal malformation rates for each item were calculated; e) donor sperm and donor egg cycles. The age at treatment for infertility, number of cycles, clinical pregnancy rates, miscarriage rates, ectopic pregnancy rates, and multiple pregnancy rates for each item were calculated; f) day 3 and day 5 embryos. The number of thawed cycles, cycle recovery rates, number of thawed embryos, embryo recovery rates, implantation rates, and clinical pregnancy rates for each item were calculated; g) AIH and AID. The age at treatment for infertility, number of cycles, clinical pregnancy rates, miscarriage rates, ectopic pregnancy rates, multiple pregnancy rates, sex ratio, live birth rates, and fetal malformation rates for each item were calculated. The missing data were treated as negative value. The follow-up rates were 98.71%, 99.34%, 99.25%, 99.43% and 99.54% from 2011 to 2015, respectively.

The definitions and calculations were performed according to The International Committee for Monitoring Assisted Reproductive Technology (ICMART) and the World Health Organization (WHO) Revised Glossary on ART Terminology [7]. To ensure high quality data, the patients, clinical, laboratory, and follow-up information were verified by the members of the expert committee. In addition, an independent retrospective survey was organized by the experts to find deficiencies and inaccuracies in the data. This study was conducted in compliance with local and national regulations and was approved by the Institutional Review Board of Liaoning Provincial Health and Family Planning Commission.

### Statistical analysis

Statistical analysis of the time trends in each data set from 2012 to 2016 were evaluated by linear regression analysis and were considered significant at *p* < 0.05.

## Results

### Characteristics and trends of ART and clinical pregnancy rates

**Fig. 1:**
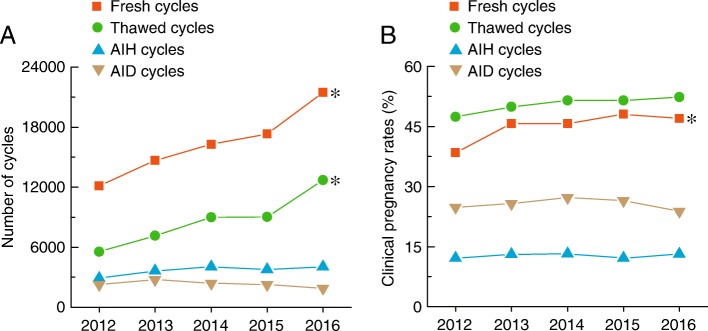
Type of ART and clinical pregnancy rates in Liaoning province from 2012 to 2016. **a** and **b**, total number of cycles and clinical pregnancy rates in fresh, thawed, AIH, and AID cycles in Liaoning province from 2012 to 2016. * *p* < 0.05

Figure 1 shows the characteristics of ART and clinical pregnancy rates in Liaoning province during the 5-year observational period. From 2012 to 2016, there was a significant increase in the number of fresh and thawed cycles (Fig. 1a), and there were also increasing trends of clinical pregnancy rates for both fresh and thawed cycles (Fig. 1b). In addition, there were significant differences in the number of cycles and clinical pregnancy rates between intrauterine insemination with husband semen (AIH) cycles and intrauterine insemination with donor semen (AID) cycles in Liaoning province from 2012 to 2016 (Fig. 1a and b).

### Characteristics and trends of ART patients

**Fig. 2:**
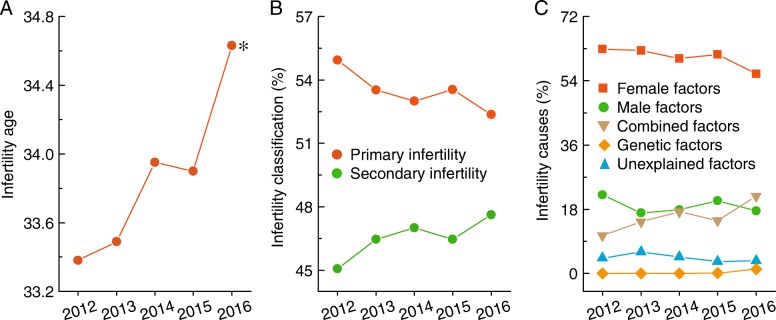
Baseline characteristics of ART patients in Liaoning province from 2012 to 2016. **a**–**c**, The age at treatment for infertility, classification, and causes are shown in Liaoning province from 2012 to 2016. * *p* < 0.05

Notably, the age at treatment for infertility gradually increased over time (Fig. 2a). In addition, the proportion of primary infertility decreased, but the proportion of secondary infertility increased during the observational period (Fig. 2b). Female, male, and combined factors were the three majority causes of infertility (Fig. 2c).

### Comparison of the features of different ovarian stimulation protocols

As shown in Table 1, standard long gonadotropin-releasing hormone (GnRH) agonists were the major ovarian stimulation protocol in Liaoning province from 2012 to 2016. During the observation period, increasing trends were mainly observed for GnRH antagonists, mild stimulation, and natural cycles. However, a decreasing trend was observed for short GnRH agonist protocols. For the number of retrieved oocytes, a decreasing trend was observed in both short GnRH agonist and mild stimulation protocols. In contrast, increasing trends were observed in super long GnRH agonist protocols. Of note, standard long GnRH agonist protocols showed the highest number of retrieved oocytes, implantation rates, and clinical pregnancy rates compared with other protocols. In addition, for early miscarriage rates, we only observed a decreasing trend for super long GnRH agonist protocols. For ectopic pregnancy and multiple pregnancy rates, we observed decreasing trends for mild stimulation and GnRH antagonist protocols, respectively. The highest dose of gonadotropin was used in the super long GnRH agonist group. In contrast, gonadotropin was rarely used in natural cycle procedures. Other protocols used a moderate dose of gonadotropin.

**Table 1:** Comparison of the features of different ovarian stimulation protocol

Protocol Year	Super long GnRH agonist	Standard long GnRH agonist	Short GnRH agonist	GnRH antagonist	Mild stimulation	Natural cycle
Percentage of different ovarian stimulation protocol (%)						
2012	4.38	58.31	22.57	8.30	4.93	1.51
2013	4.03	68.77	11.25	6.19	6.81	2.94
2014	4.71	62.65	13.51	4.95	10.23	3.95
2015	4.67	53.59	8.40	15.16	13.61	4.58
2016	6.24	43.69	4.60*	26.39	12.89*	4.65*
Number of retrieved oocytes						
2012	7.63	11.94	8.27	6.29	3.50	0.93
2013	9.99	11.42	7.69	6.42	2.80	0.82
2014	8.41	11.30	7.45	5.69	2.43	0.69
2015	10.87	11.04	7.23	6.31	2.32	0.72
2016	13.00	12.00	6.03*	6.70	2.24*	0.73
Implantation rates (%)						
2012	27.53	31.58	22.22	17.65	12.26	18.60
2013	29.07	31.18	20.67	21.74	16.19	18.89
2014	33.64	33.17	23.71	20.86	15.06	22.73
2015	33.94	34.65	25.14	24.47	17.42	18.33
2016	31.21	34.60*	25.58	25.11*	17.01	21.97
Clinical pregnancy rates (%)						
2012	45.59	48.56	33.42	29.27	18.34	26.03
2013	49.26	49.68	33.25	36.70	24.72	18.99
2014	53.63	49.77	37.58	35.22	22.85	21.30
2015	49.75	53.14	41.75	39.01	31.33	20.75
2016	49.44	52.90*	42.32*	39.41	25.69	22.73
Early abortion rates (%)						
2012	12.44	11.79	14.79	19.74	24.66	26.32
2013	8.58	7.42	6.44	10.00	10.91	0.00
2014	9.41	10.68	9.98	15.52	15.38	8.70
2015	9.09	7.63	6.64	11.15	11.96	0.00
2016	8.63	8.53	11.31	9.05	14.88	16.00
Ectopic pregnancy rates (%)						
2012	1.84	2.94	2.47	3.86	4.11	0.00
2013	4.29	2.40	4.46	10.50	3.64	13.33
2014	1.76	1.62	3.21	4.60	3.08	0.00
2015	1.35	2.29	3.65	2.69	0.48	4.55
2016	1.52	2.72	0.60	3.34	1.65	0.00
Multiple pregnancy rates (%)						
2012	22.58	27.95	27.26	24.46	13.70	0.00
2013	32.62	26.09	24.01	24.00	18.18	13.33
2014	26.76	31.19	32.26	21.26	16.15	8.70
2015	35.35	31.48	31.23	22.12	6.70	4.55
2016	20.05	27.55	23.21	21.66	14.05	16.00
Average dose of gonadotropin (IU)						
2012	3101.25	2356.5	2251.5	2379.75	1019.25	0
2013	3190.5	2541	2342.25	2201.25	945.75	9
2014	3342.75	2598	2406	2372.25	852.75	0
2015	3192	2560.5	2379	2419.5	913.5	9
2016	2678.25	2461.5	2418.75	2347.5	1176	37.5

*p < 0.05, represents a significant time trend in the each data from 2012 to 2016

### Comparison of the features of conventional IVF and ICSI

**Fig. 3:**
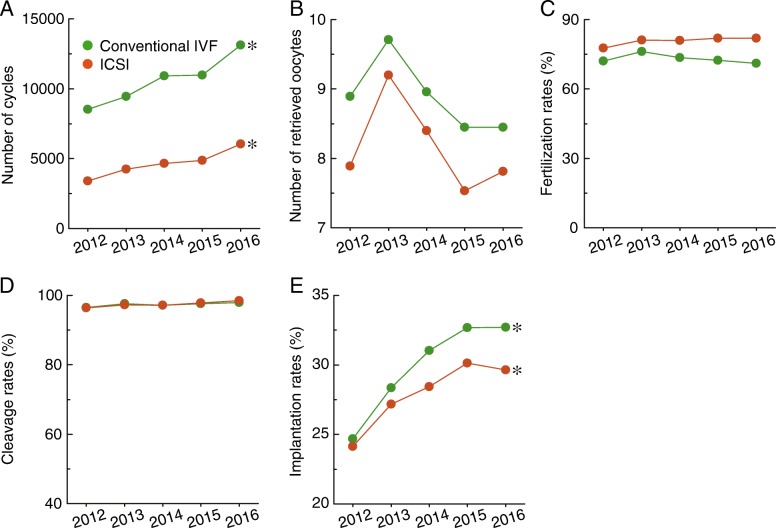
Comparison of the features of conventional IVF and ICSI. **a**–**e**, The number of cycles, number of retrieved oocytes, fertilization rates, cleavage rates, and implantation rates are shown for conventional IVF and ICSI in Liaoning province from 2012 to 2016. * *p* < 0.05

Significant differences in the number of cycles (Fig. 3a), number of retrieved oocytes (Fig. 3b), fertilization rates (Fig. 3c), and implantation rates (Fig. 3e) were noticed between conventional IVF and ICSI. No significant differences were observed in cleavage rates between conventional IVF and ICSI (Fig. 3d). In addition, increasing trends in number of cycles (Fig. 3a) and implantation rates (Fig. 3e) were observed for both of these methods.

### Comparison of the features of donor sperm cycles and donor egg cycles

**Fig. 4:**
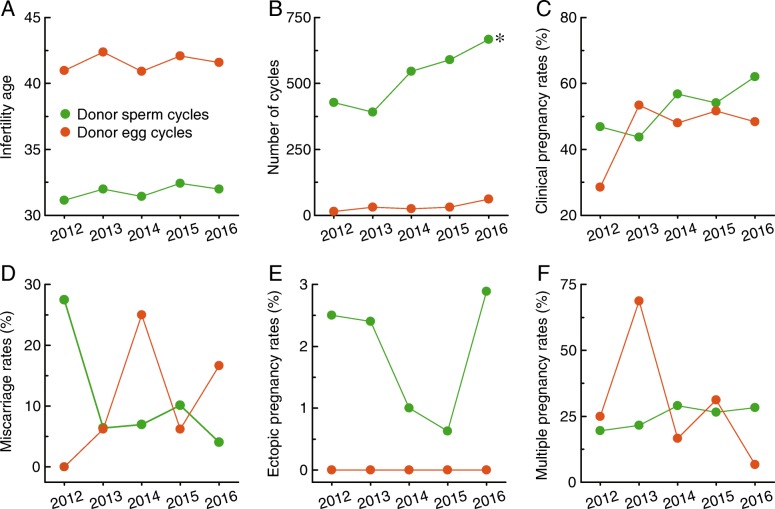
Comparison of the features of donor sperm cycles and donor egg cycles. **a**–**f**, The age at treatment for infertility, number of cycles, clinical pregnancy rates, miscarriage rates, ectopic pregnancy rates, and multiple pregnancy rates are shown for donor sperm cycles and donor egg cycles in Liaoning province from 2012 to 2016. * *p* < 0.05

Significant differences in age at treatment for infertility (Fig. 4a), number of cycles (Fig. 4b), and ectopic pregnancy rates (Fig. 4e) were noticed between donor sperm cycles and donor egg cycles. There were increasing trends of clinical pregnancy rates (Fig. 4c) and multiple pregnancy rates (Fig. 4f) and decreasing trends of miscarriage rates (Fig. 4d) in donor sperm cycles, but no significant differences were observed in clinical pregnancy rates (Fig. 4c), miscarriage rates (Fig. 4D), or multiple pregnancy rates (Fig. 4f) between donor sperm cycles and donor egg cycles.

### Comparison of the features of thawed cycles

**Fig. 5:**
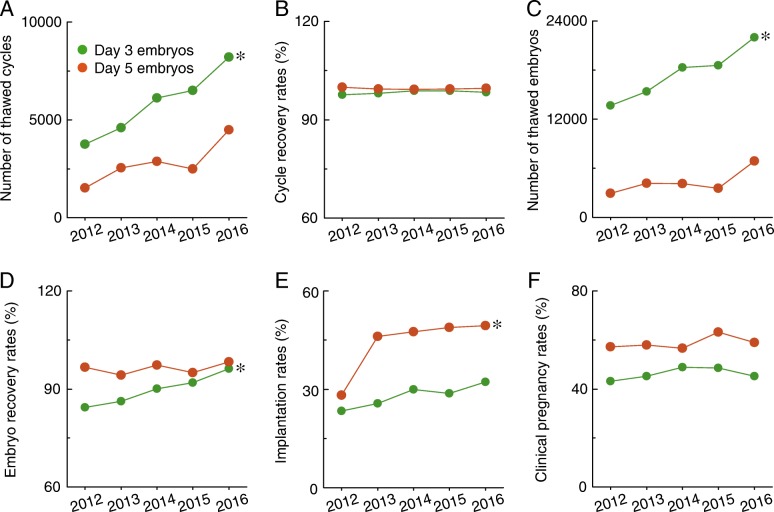
Comparison of the features of thawed cycles. **a**–**f**, The number of thawed cycles, cycle recovery rates, number of thawed embryos, embryo recovery rates, implantation rates, and clinical pregnancy rates are shown for day 3 embryos and day 5 embryos in thawed cycles in Liaoning province from 2012 to 2016. * *p* < 0.05

Significant differences in the number of thawed cycles (Fig. 5a), number of thawed embryos (Fig. 5c), embryo recovery rates (Fig. 5d), implantation rates (Fig. 5e), and clinical pregnancy rates (Fig. 5f) were noticed between day 3 and day 5 embryos. No significant differences were observed in cycle recovery rates between day 3 and day 5 embryos (Fig. 3b). Of note, there were significantly increased numbers of thawed cycles (Fig. 5a), thawed embryos (Fig. 5b), and implantation rates (Fig. 5e) for both of these methods in Liaoning province from 2012 to 2016.

### Comparison of the features of AIH and AID

**Fig. 6:**
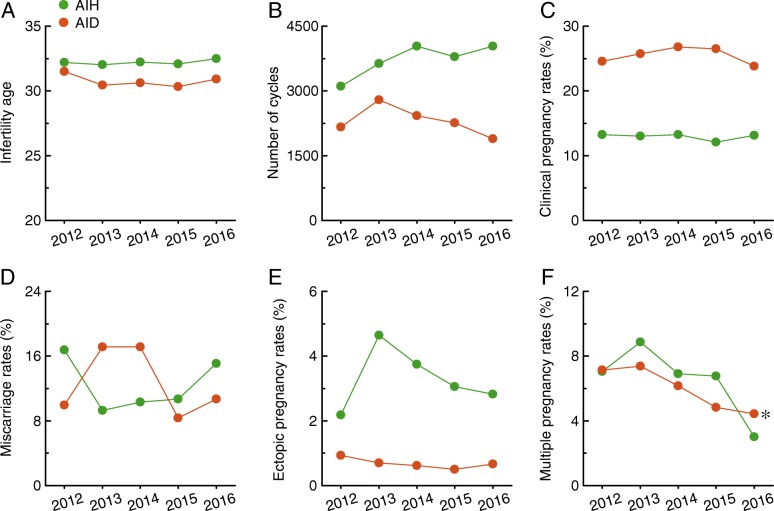
Comparison of the features of AIH and AID. **a**–**f**, The age at treatment for infertility, number of cycles, clinical pregnancy rates, miscarriage rates, ectopic pregnancy rates, and multiple pregnancy rates are shown for AIH and AID in Liaoning province from 2012 to 2016. * *p* < 0.05

Significant differences in age at treatment for infertility (Fig. 6a), number of cycles (Fig. 6b), clinical pregnancy rates (Fig. 6c), and ectopic pregnancy rates (Fig. 6e) were noticed between AIH and AID. An increasing trend was only observed for the number of cycles for AIH (Fig. 6b). However, a decreasing trend in multiple pregnancy rates (Fig. 6f) was observed for both of these methods. No significant differences between AIH and AID were observed for miscarriage rates (Fig. 6d) or multiple pregnancy rates (Fig. 6f).

### Comparison of clinical outcomes of pregnancy between conventional IVF and ICSI with AIH and AID

**Fig. 7:**
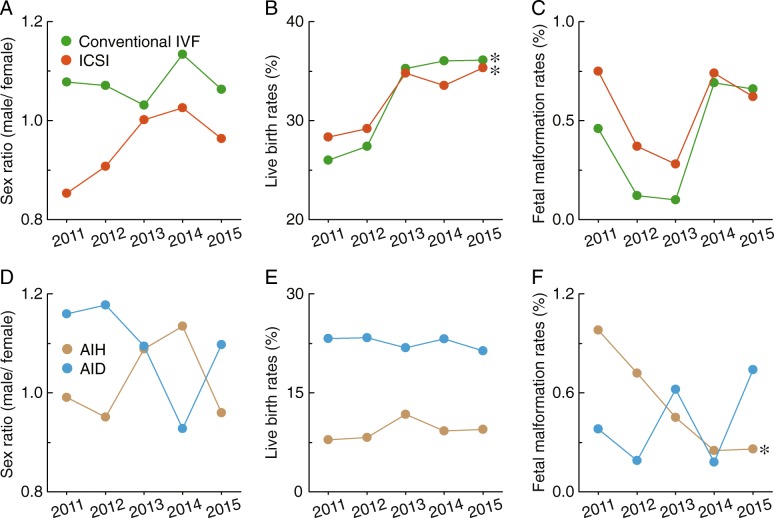
Comparison of clinical outcomes of pregnancy for conventional IVF and ICSI, or AIH and AID. The sex ratio (**a** and **d**), live birth rates (**b** and **e**), and fetal malformation rates (**c** and **f**) are shown for conventional IVF and ICSI or AIH and AID in Liaoning province from 2012 to 2016. * *p* < 0.05

A significant difference in the sex ratio was noticed in a comparison of conventional IVF and ICSI (Fig. 7a) but not between AIH and AID (Fig. 7d). Similarly, significant differences in the live birth ratio were observed between AIH and AID (Fig. 7e). An increasing trend of live births was observed for both conventional IVF and ICSI, although there was no significant difference between these two groups (Fig. 7b). In addition, a decreasing trend of fetal malformation rates was only observed in populations receiving AIH (Fig. 7f), with no significant difference between conventional IVF and ICSI (Fig. 7c).

## Discussion

To date, no studies have reported detailed ART data from China. To our knowledge, this is the first comprehensive study describing and analysing the ART technical status from clinics and IVF laboratories in the largest province in the northeast of China.

It is well-known that environmental toxins, food additives, medications, unhealthy lifestyles, and mental stress contribute to the increasing incidence of infertility in China [2]. Consistent with this, there was a significant increase in the number of fresh cycles (from 12,143 to 21,448) and thawed cycles (from 5542 to 12,697) in Liaoning province from 2012 to 2016. Moreover, as of December 31, 2012, the National Health and Family Planning Commission of the People’s Republic of China had authorized 365 organizations to conduct ART treatment [8]. In just 4 years, the number of approved organizations has increased to 451 as of December 31, 2016 [9]. These observations suggest that infertility is becoming a serious health problem in China.

In addition, taking the data presented here into consideration, it is interesting to note that the proportion of cases of primary infertility has gradually decreased, but secondary infertility gradually increased in Liaoning province from 2012 to 2016, and female factors were the major cause of infertility (~ 60% of cases). This suggests that women’s lifestyles are undergoing great changes in China, and may play a critical role in infertility. Consistent with this, an emerging body of evidence indicates that lifestyle modification (behavioral management and dietary and exercise interventions) may be used as the first line of ovulation induction in PCOS patients [10], and lifestyle intervention leads to increased natural conception in anovulatory obese women [11]. Although the exact reasons for the increases in secondary infertility are not fully understood, China’s new ‘two-child policy’ may be responsible for the increasing trend of secondary infertility, due to older mothers and more cases of secondary infertility being diagnosed. In addition, it has been suggested that cocaine, marijuana and alcohol use, exercise, caffeine consumption, and over-use of thyroid medications are possible risk factors for various subtypes of primary infertility [12]. Standard long GnRH agonist was the major ovarian stimulation protocol in Liaoning province in the past, but the proportion of mild stimulation and natural cycles has gradually increased from 2012 to 2016. It seems that age of being treated for infertility has gradually increased over time, and people are increasingly inclined to accept natural stimulation protocols. Although the number of ART cycles has been increasing, the ratio of conventional IVF cycles to ICSI cycles has remained constant at about 2.3 from 2012 to 2016. These data indicate that the overuse of ICSI technology is tightly controlled in Liaoning province. Importantly, the average age of being treated with donor egg cycles and donor sperm cycles is 41.58 and 31.79, respectively. The average number of donor sperm cycles is about 16 times greater than donor egg cycles, and the number of donor sperm cycles is growing rapidly, but the number of donor egg cycles has not increased significantly. Donor eggs are still a scarce resource in China. In response to the growing need for donor eggs, a donor egg bank should be established and perfected, to facilitate the treatment process and assist infertility patients in their desire to conceive. Notably, despite the declining trend of multiple pregnancy rates, they still remain around 20% for most treatments. Take the standard long protocol as an example, the average multiple pregnancy rate from 2012 to 2016 was 28.85%, and the number of embryo transfers per treatment was 2.05 ± 0.07 in the past five years, which could explain the high multiple pregnancy rate. We will reduce the number of embryo transfers in an effort to curtail the multiple pregnancy rate.

Although this is the first study to describe and analyze the ART technical status in China, some limitations of our study should be acknowledged. First, the data only considered patients over a relatively short period (5 years) in Liaoning province, and therefore lacked information about ART technical status in other provinces and study periods. Thus, we failed to compare the ART situations across other areas in China. Second, we could not exclude the possibility that registration problems might exist for the data used in our study. The study had a retrospective design, which might have introduced recall or information bias into our data. Finally, we did not carry out any subgroup analyses based on some potential confounders because of the limitations of the data. It seems that some women might be in different situations due to different educational levels or socioeconomic status. However, we could not detect the influence of potential confounders to ART in our study.

Public health surveillance is the continuous collection and interpretation of outcome-based data for the purpose of use in public health practice. We will continue to collect surveillance data and use it to identify problems and assess the effectiveness of ART-related interventions, especially the live birth rates, clinical pregnancy rates, multiple pregnancy rates, sex ratio, fetal malformation rates. China‘s family planning policy, which was first announced in 1979, aimed to control population growth [13]. Interestingly, ART in China has experienced a tremendous evolution since the first IVF infant was born in 1988, suggesting that ART is becoming an important method, alongside contraception, sterilization, and abortion, in cooperatively maintaining a balanced population [14]. However, it should be recognized that ART is not a perfect technology, and there is constant debate about supporting or opposing the use of ART.

## Conclusions

ART in Liaoning province has undergone substantial development from 2012 to 2016 in clinics and IVF laboratories. This presentation of detailed ART data can help researchers, policy makers, and potential ART users to better understand the technology.

## Abbreviations

AID: Intrauterine insemination with donor semen
AIH: Intrauterine insemination with husband semen
ART: Assisted reproduction techniques
GnRH: Gonadotropin-releasing hormone
ICSI: Intracytoplasmic sperm injection
IVF: In vitro fertilization
